# Inferring Anomaly Situation from Multiple Data Sources in Cyber Physical Systems

**DOI:** 10.1007/978-3-030-69781-5_5

**Published:** 2021-01-28

**Authors:** Sara Baldoni, Giuseppe Celozzi, Alessandro Neri, Marco Carli, Federica Battisti

**Affiliations:** 8 Norwegian Computing Center, Oslo, Norway; 9 University of Trento and Fondazione Bruno Kessler, Trento, Italy; 10 Università degli Studi di Genova, Genoa, Italy; 11 IEIIT Institute, Consiglio Nazionale delle Ricerche (CNR), Genoa, Italy; 12 SINTEF A.S., Oslo, Norway; 13 Engineering Ingegneria Informatica S.p.A., Rome, Italy; 14 Instituto Superior de Engenharia do Porto, Porto, Portugal; 15 University of Padua, Padua, Italy; 16 Roma Tre University, Rome, Italy; 17 Ericsson S.p.A., Rome, Italy

**Keywords:** Anomaly detection, Cyber physical system, Critical infrastructure protection

## Abstract

Cyber physical systems are becoming ubiquitous devices in many fields thus creating the need for effective security measures. We propose to exploit their intrinsic dependency on the environment in which they are deployed to detect and mitigate anomalies. To do so, sensor measurements, network metrics, and contextual information are fused in a unified security architecture. In this paper, the model of the proposed framework is presented and a first proof of concept involving a telecommunication infrastructure case study is provided.

## Introduction

Cyber Physical Systems (CPSs) can be defined as the result of the integration of computing, communication, and control capabilities for monitoring and managing physical world objects [5]. The “Industry 4.0” paradigm is pushing the spread of CPSs in many fields: smart manufacturing, e-health, smart city, smart vehicles, wearable devices, telecommunication systems, defense systems, etc. As can be easily understood, the security of CPSs is an open and critical issue [5].

A CPS can be described with a three-layer architecture: perception, transmission, and application. The first layer is responsible for data collection in real-time, the second deals with data exchange, and the third layer provides data processing and control functionalities. Even if the security can be addressed for each layer separately, to counteract the attacks in an effective manner, a multi-layer approach can be beneficial. The intrinsic dependency between CPSs and the sensing environment can further expand the attack surface. However, this connection can also be exploited to design effective security measures. The idea of context aware security has been introduced in [14], where the context is defined as “the set of environmental states and settings that either determine an application’s behaviour or in which an application event occurs”. Moreover, four context classes are introduced: system, user, physical environment, and time. This concept has been applied to CPSs in [8], where context is considered as a new class of information to be exploited for improving the safety and security of CPSs. The authors suggest that contextual data can be used both for inferring information about the system state, and for preventing wrong detection decisions due to bad-data scenarios. In [7], moreover, a general introduction about CPS security issues is provided together with the presentation of a context aware biometric security framework. The proposed approach fuses real-time mechanisms with contextual information such as the client setting area, lighting, temperature, climate and time. Context is considered also in the security infrastructure for Internet of Things (IoT) systems proposed in [11]. The presented architecture includes some contextual information, such as the amount and rate of collected data, which may be correlated to security indicators. Furthermore, the environmental impact has been exploited in [12], where device fingerprinting for IoT authentication is analyzed. More specifically, the authors propose to exploit the environmental effects on IoT fingerprints to detect emulation attacks. The idea is that an attacker will not be able to imitate the true environmental changes experienced by the legitimate device thus failing in reproducing an environment-based fingerprint. At last, in [10], the concept of context aware intrusion detection systems is realized by including the operating environment information in the collected data. This category involves for instance networking conditions (e.g. start and goal address/port, access frequency, and data traffic), and systematic operation conditions (e.g. the presence of idle CPU or memory occupation conditions). In this paper, we focus on the impact of the physical environment context to design a CPS anomaly detection framework, and we apply the introduced model to a telecommunication infrastructure case study. More specifically, according to [4], here we define an anomalous situation as a malicious or a genuine but unusual behaviour of the system. We propose the CPS Context Aware Security Protection for Enhanced Robustness (CCASPER) model, to identify anomalies in the system behaviour exploiting both the physical aspect of the CPS and the information that can be collected through their networking capabilities. In more details, sensor outputs, network performance indicators, and the surrounding context conditions are fused together for selecting the mitigation strategy. A multi-metric approach which treats each metric independently, in fact, would ignore the possible correlations or cause-effect relationships between them, thus resulting less effective [2].

The rest of the paper is organized as follows. In Sect. 2 the security framework is presented, in Sect. 3 the model is applied to a communication infrastructure scenario, and in Sect. 4 the conclusions are drawn.

## Proposed Method

**Fig. 1.**
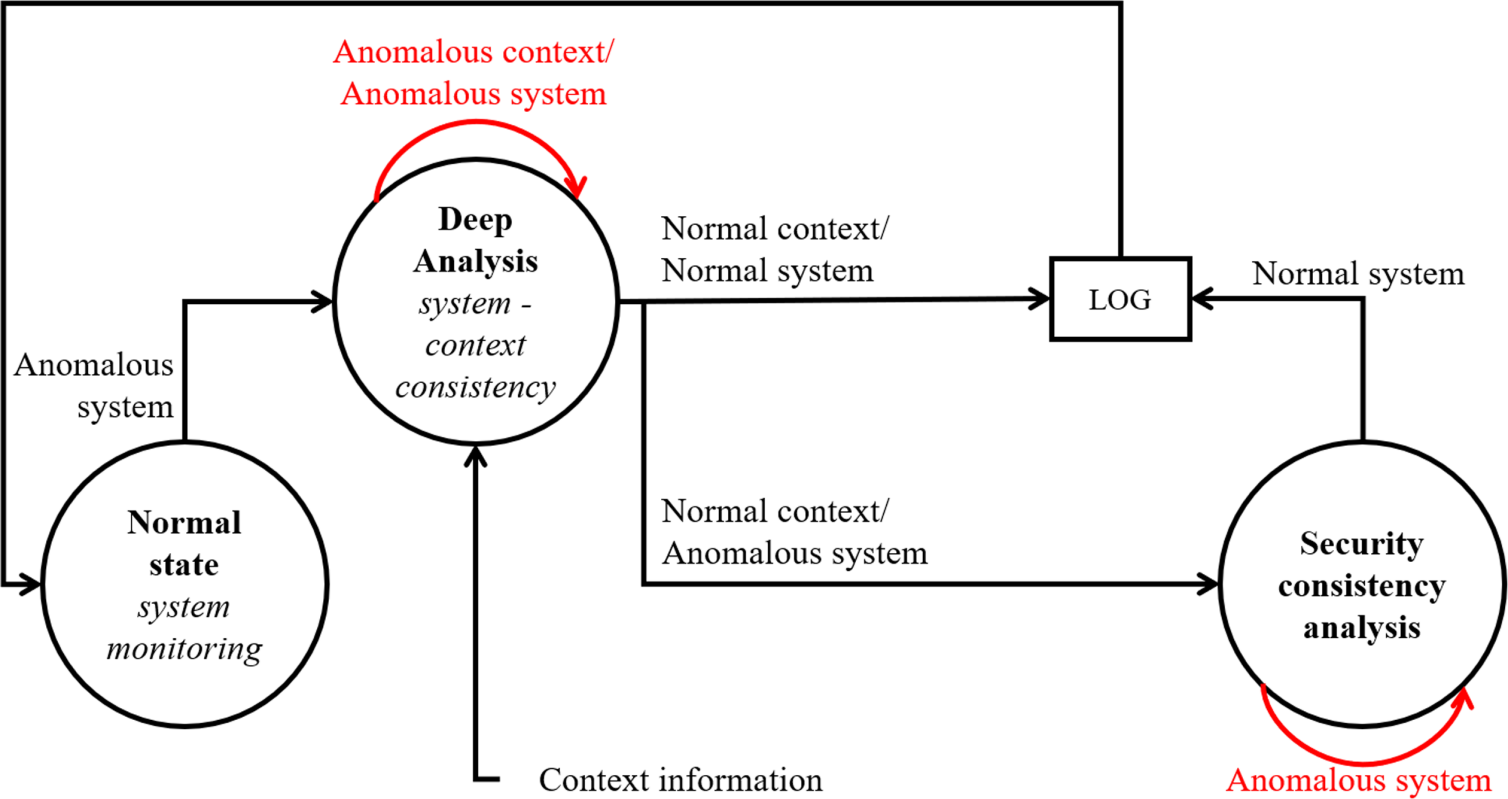
Security framework: state machine model.

The proposed CCASPER model is based on the fusion of the information gathered from two sources: the system (i.e. the deployed CPSs) and the context. Concerning the first source, information may be collected both in the perception layer (which gathers data about a physical process) and in the communication layer (which allows to share information). Nominal value ranges for sensor outputs and network performance indicators may be defined, so that when the measurements deviate from their nominal values, an anomaly is detected and an alarm is triggered. However, using only system indicators could give a partial representation of the real scenario leading to a misclassification of anomalies. The physical process and the communication link, in fact, are considerably influenced by the surrounding context. If sensor outputs and network performance indicators do not match the expected values, then, at least four causes should be investigated: a fault, a cyber-attack, or a physical attack may be occurring, or the surrounding ecosystem may be affecting network performances and/or sensor measurement. Therefore, before triggering an attack alarm, it is worth to collect contextual information to verify if a natural event (a storm, an earthquake, or strong wind) may cause such anomaly. To this aim, the proposed model relies on a context monitoring algorithm localized around the CPS position. In fact, adverse environment conditions may cause a temporary decrease of network performance or, even, damages to the physical system (e.g., antenna collapsing or broken sensor). In the first case, once the environmental emergency is over, the normal operating level of the network has to be quickly restored. On the contrary, a physically damaged system must be repaired. The proposed security model can be described through the state machine architecture shown in Fig. 1.

The state machine model is detailed as follows. In the *normal state* the system monitoring is performed through the analysis of application-dependent quantitative attributes. For example, in a point to point connection, network performance metrics could be delay or throughput values while the sensor measurements could be temperature, humidity, or antenna tilt. If a relevant deviation from the nominal behavior occurs, the model moves to the *deep analysis state*. In this state, a first consistency analysis is performed to verify if the system behaviour can be justified through the current context conditions. To do so, several additional information sources can be analysed: weather news, local news, exceptional events such as flooding or fire, etc. Moreover, due to the distributed nature of CPSs, information gathered from neighbouring devices can be included in the consistency analysis. The context, in fact, should cause a similar deviation from the nominal behaviour for CPSs deployed on the same area. The gathered data are exploited to perform the consistency check between the current context and the quantitative attributes, leading to one of the following outcomes:

- anomalous context/anomalous system: the environmental context may have caused a temporary network performance decrease or a change in the monitored physical process so that the measured values deviate from the nominal range. In this case a joint monitoring is performed until when the context anomalous behaviour is over. Two outcomes are possible:
  - the system returns in the nominal range: the event is saved in a log file, and the system goes back to the *normal state*.
  - the system anomaly persists thus requiring a further analysis. The model moves to the *security consistency analysis state*.
- normal context/anomalous system: a further analysis is needed, the model goes in the *security consistency analysis state*.

**Fig. 2.**
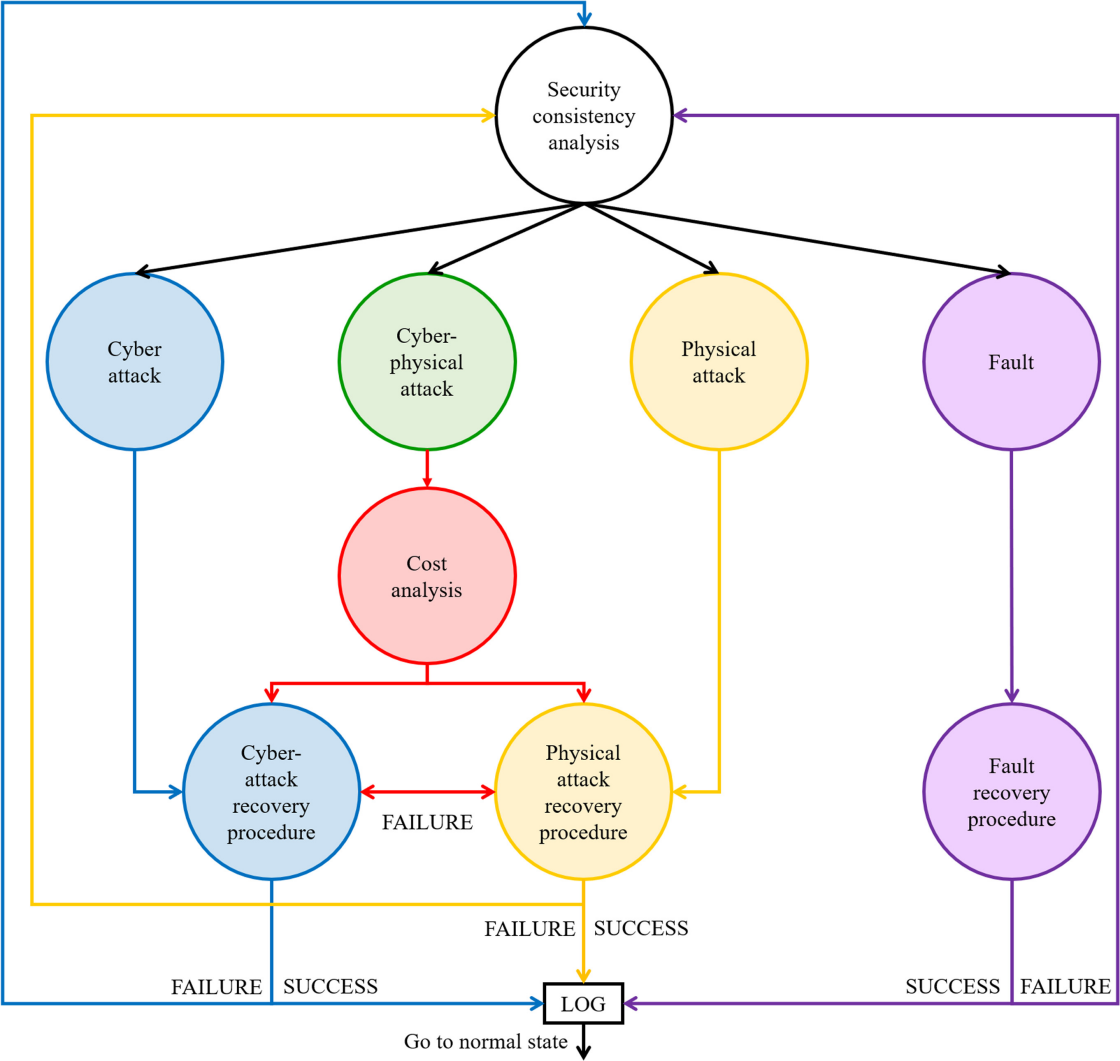
Security framework: consistency analysis model.

If the model goes in the *security consistency analysis state* an additional monitoring is performed in order to identify the possible causes for system anomaly. This state can be further expanded as shown in Fig. 2.

Depending on the use case, different combinations of the monitored parameters may lead to four states which, in turn, represent the anomaly cause:

- **cyber attack** a cyber attack to the sensor may be on going;
- **cyber-physical attack** an attack involving both physical and cyber aspects of the system may be on going.
- **physical attack** a physical damage of the system may have occurred;
- **fault** a system fault (e.g. a component damage) may have occurred.

Once the cause has been identified, the associated recovery procedure is deployed. If such procedure is effective, the event occurrence is saved and the model returns to the *normal state*, otherwise a new security consistency check is performed. Let us note that in case of cyber-physical attacks, a further study is needed to select the most suitable recovery procedure. As first proof of concept, here we present the case in which the choice between cyber and physical recovery plans is performed based on cost. However, depending on the application, other parameters could be considered such as the time needed for the solution deployment. In some scenarios, in fact, restoring the system availability is the most crucial issue.

## Case Study

In this paper we deal with a use case scenario involving a mobile network operator radio infrastructure. The strict 5G requirements lead to the need of relevant improvements in the Radio Access Network (RAN). To this aim, the classic rule-based network functionalities can be replaced by their Artificial Intelligence (AI) based counterparts. However, a key enabler for the application of AI in this context is a deep insight into the nature and role of the different network performance contributors [1]. Moreover, the satellite segment of 5G networks is one of the main topics in the 5G development in 2020–2025 [13] and, as highlighted in [9], for such applications the antenna pointing and the mobile tracking are crucial. More specifically, for pointing purposes, the AI-based method proposed in [9] exploits data acquired by the inclinometer and an electronic compass. In addition, antenna tilting is considered as a key enabler for RAN optimization also in [1]. Let us note that tilt monitoring is of utmost importance both for mechanical and electronic tilting systems. In the former case it is needed to correctly set the beam pointing, whereas in the latter the physical orientation of the antenna has to be set and kept with sufficient accuracy. For this reason, we included a tilt sensor installed on the base station antennas in our case study.

**Fig. 3.**
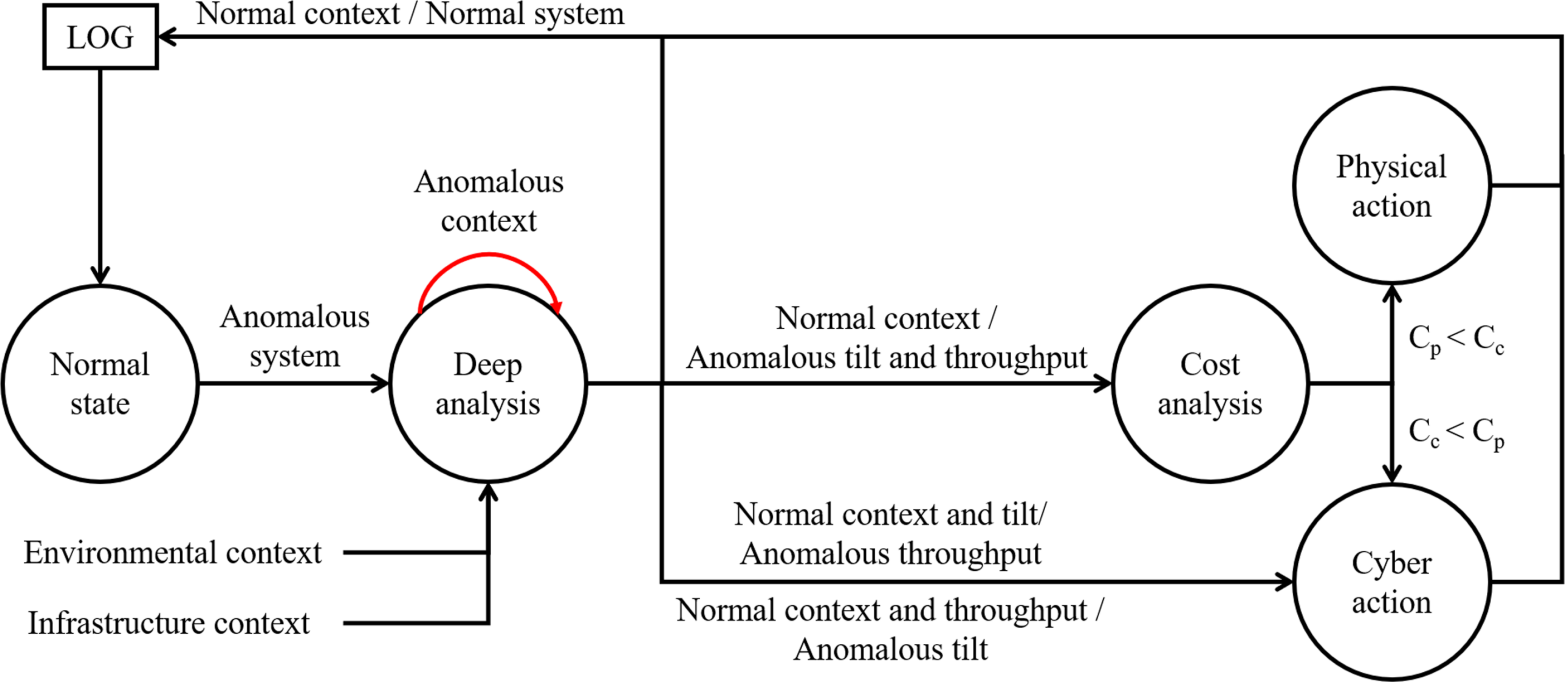
Security framework: case study model. In the figure, $C_c$ and $C_p$ represent the cyber and physical action cost, respectively.

Concerning the network performance metrics, we referred to the security studies performed on the existing LTE infrastructure. In [6], for instance, the authors analyzed three metrics: throughput, end to end delay, and packet delivery rate. Their simulations show a significant impact on these parameters due to malicious user equipment devices, malicious base stations, malicious connections, and malicious femtocells. To show the operating principle of the proposed framework, in this work we considered only the throughput, but the analysis can be extended to the other network parameters as well. In this case, the general framework presented in Fig. 1 can be modified as shown in Fig. 3.

As in the general model, once an anomalous parameter is detected, the system moves to the *deep analysis state*. More in details, we included in the context monitoring the analysis of: weather, terrorism, civil protection department alerts, earthquakes, floods, severe storm, and fire. Moreover, in this scenario, the measurements collected by nearby base stations can be considered as an additional information source. As a consequence, the context information includes both the surrounding environment and the neighbouring infrastructure. The deep analysis correlates these data with the current quantitative attributes. For instance, a heavy rain may cause a severe attenuation phenomenon which, in turn, causes a throughput reduction without any impact on the antenna tilting. A strong wind, on the contrary, may cause an antenna movement which will produce a pointing inaccuracy. In this case, the first phenomenon will be detected by the tilt sensor, whereas the latter will have an impact on the measured throughput. Under these circumstances, the system stays in the *deep analysis state* until when the environmental emergency is over.

If the contextual cause can be excluded, or if the system parameters are still anomalous when the context emergency is over, a consistency check of the remaining inputs is performed. The analysis of the throughput indicator and the antenna tilt sensor, in fact, can lead to different scenarios. A normal tilt value in conjunction with an anomalous throughput can suggest a channel attack. The opposite situation can be due to a cyber-attack to the tilt sensor. If both indicators fail to satisfy their requirements, a physical attack to the antenna, or the combination of a channel and sensor attacks, should be considered. Moreover, the anomalous context may have caused a network damage which has to be physically repaired. If both cyber and physical recovery hypothesis need to be checked, the mitigation scheduling is chosen according to the cost of the required operations ($C_c$ and $C_p$ respectively). As a consequence, the cheaper recovery procedure is deployed first and, if it is not effective, the other one is performed. Let us note that when a “Physical action” is required, a technical team will be sent to the antenna location. This solution will thus be required in case of physical antenna damage.

**Fig. 4.**
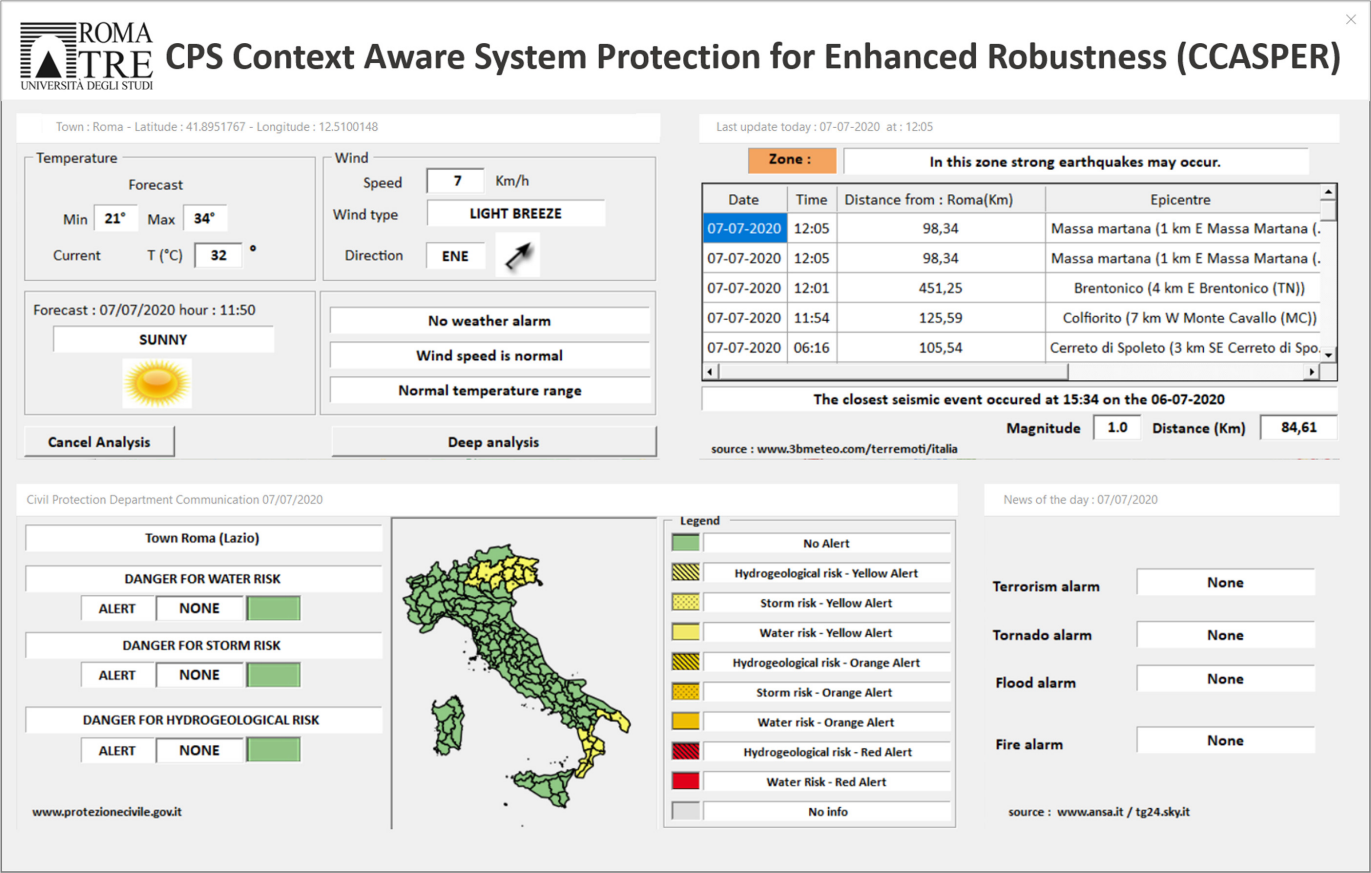
Context data collection: the CCASPER GUI

As first proof of concept of the proposed security framework we implemented the CCASPER model and a user interface. In order to gather up-to-date environmental context information, the antenna position has to be retrieved. To this aim, we used the antenna map provided in [3]. Moreover, in the implemented system, antennas can be deleted or added directly from the Graphical User Interface (GUI). From the tool GUI, it is possible to select a specific antenna and run the simulation. In the performed tests, as a proof of concept, tilt and throughput data have been entered by the user. The implementation of the complete framework which collects the tilt information from the sensor and uses the measured throughput will be the object of future research. Once the tilt and throughput data have been entered, context information about the location where the antenna is placed is collected and shown as presented in Fig. 4. The button “Deep analysis” allows to move to the corresponding state in which, as first step, the context analysis is performed. In case of normal environmental conditions, according to the tilt and throughput values, a cost analysis or a cyber action can be suggested as shown in Fig. 3.

**Fig. 5.**
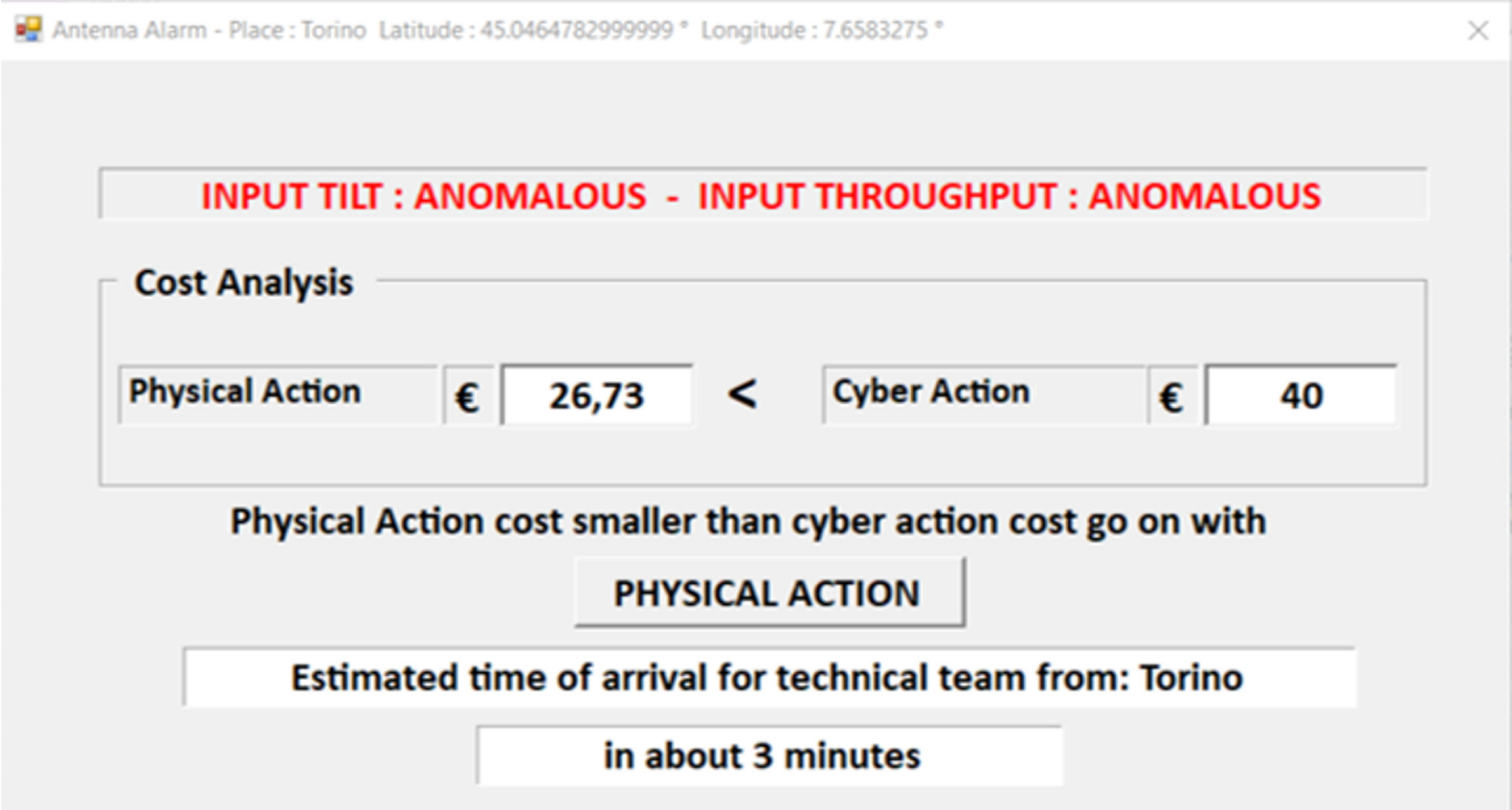
Cost analysis output.

Concerning the cost analysis, the physical action cost may be computed depending on the distance of the antenna from the actual position of the technical team. More specifically, we assumed to have an office for each town and we considered a total cost made of a fixed component plus a variable cost linked to the distance from the antenna. However, it is possible to configure the costs directly from the GUI main page. An example of the cost analysis output is provided in Fig. 5.

The proposed model allows to process the information coming from several data sources to figure out the reason behind an anomalous behaviour of the system, thus suggesting a suitable mitigation strategy. Although limited to the analysis of two quantitative attributes, the presented approach can be easily extended for including other performance indicators (e.g. delay) and/or other sensor measurements (e.g. temperature). According to the analyzed case study, we argue that the fusion of environmental, technical, and financial information sources is a key enabler to provide prompt reactions to communication infrastructure anomalies.

## Conclusions

In this contribution, a model for inferring anomaly situations is proposed. Technical and environmental inputs are considered for defining the best mitigation technique in terms of effectiveness and financial feasibility. We implemented the proposed security framework through a state machine model which provides the general architecture of the proposed approach. As first proof of concept we analyzed a telecommunication infrastructure case study. To do so, we realized a tool for collecting and fusing the information coming from the different sources, and exploited a user friendly graphical interface for entering input data.

The proposed framework can be applied to every scenario involving CPSs, after the definition of application-dependent quantitative attributes. We believe that the fusion of several information sources is a crucial facilitator for inferring anomalous situations in CPS-based critical infrastructures.
